# Risk Factors and Coinfection Dynamics of Pathogens in Wild Turkeys (*Meleagris gallopavo*) From Pennsylvania, USA

**DOI:** 10.1002/ece3.73079

**Published:** 2026-02-10

**Authors:** Ryan W. Koch, Axel O. G. Hoarau, Tryssa de Ruyter, Caitlin Duffy, Lucie Pascarosa, Kerry A. Campbell, Casey L. Maynard, Andrew Cushman, Heather Flick, Anthony Musselman, Julianna Patsko, Rachel Bealer, Graham Rhone, Mary Jo Casalena, Andrew Di Salvo, Ken Duren, Jay T. Armstrong, Frances E. Buderman, R. Scott Larsen, Caroline Sobotyk, Erica A. Miller, Kevin D. Niedringhaus, Brock Geary, Eman Anis, Roderick B. Gagne

**Affiliations:** ^1^ Department of Pathobiology, Wildlife Futures Program University of Pennsylvania Kennett Square Pennsylvania USA; ^2^ Department of Veterinary Microbiology and Pathology Washington State University Pullman Washington USA; ^3^ Pennsylvania Game Commission Harrisburg Pennsylvania USA; ^4^ Department of Pathobiology University of Pennsylvania Philadelphia Pennsylvania USA; ^5^ Department of Ecosystem Science and Management Pennsylvania State University University Park Pennsylvania USA; ^6^ Department of Pathobiology, PADLS New Bolton Center University of Pennsylvania Kennett Square Pennsylvania USA

**Keywords:** disease surveillance, infectious diseases, LPDV, *Mycoplasma*, parasite, wildlife

## Abstract

Interactions between co‐occurring pathogens can have complex and significant impacts on host survival, fitness, and population dynamics. While common in wildlife, coinfections are often overlooked, and research may create biased management perspectives when individual pathogens are assessed in isolation. Recent work has found that wild turkeys ( *Meleagris gallopavo* ) are affected by various pathogens, but it is unknown how infections and coinfections are spatially structured or interact with each other. Here, we determined the associations and risk factors of infection by lymphoproliferative disease virus (LPDV), reticuloendotheliosis virus (REV), three avian *Mycoplasma* species, and internal parasites in Pennsylvania wild turkeys. Our results indicate varying prevalences: LPDV (70%), REV (1%), *Mycoplasma gallisepticum* (0%), *Mycoplasma meleagridis* (4%), *Mycoplasma synoviae* (2%), and internal parasites (63%). The prevalence of LPDV was greater in adults than juveniles but did not vary with year, sex, study area, or landscape type. Parasite species richness was greater in juveniles than adults, greater in males than females, varied by year and study area, but did not vary with landscape type. Coinfections with LPDV and parasites were more common (41%) than infections with only LPDV (26%) or only parasites (22%). All other coinfection prevalences involving viruses, *Mycoplasma* species, and parasites were low (0%–3%). Finally, infection with LPDV did not differ with overall parasite species richness but was negatively associated with infection with parasitic nematodes. These results reveal high rates of coinfections with LPDV and parasites in turkeys but suggest that parasite infections are independent of LPDV infections. Ongoing work is currently investigating the sublethal effects of these coinfections on wild turkey populations.

## Graphical abstract

We determined the associations and risk factors of infection by lymphoproliferative disease virus (LPDV), reticuloendotheliosis virus (REV), three avian *Mycoplasma* species, and internal parasites in Pennsylvania wild turkeys ( *Meleagris gallopavo* ). Active surveillance of parasites and pathogens was conducted over four years and four regions across Pennsylvania. Our results reveal high rates of coinfections with LPDV and parasites but suggest that parasite infections are independent of LPDV infections.

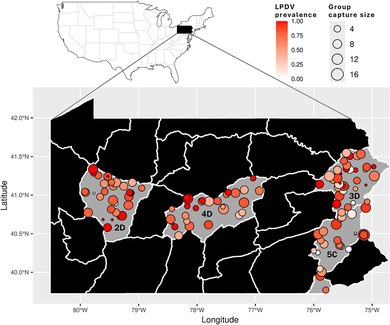

## 1 Introduction

Parasites are a natural component of wildlife populations that have complex disease outcomes on hosts as well as important ecosystem functions (Goater et al. 2014; Hudson et al. 1992, 2002, 2006; Thompson et al. 2010). For example, parasites, defined based on their dependent lifestyle on a host, can contribute to ecosystem health by regulating trophic interactions, food webs, and biodiversity, while at other times causing drastic population or species‐level declines due to severe diseases (Hudson et al. 1992, 2006; Thompson et al. 2010). Parasites can also have a variety of sub‐lethal effects on reproduction, survival, growth rates, metabolic requirements, immune investment, and behavior (Delahay et al. 2009). These effects are difficult to document in free‐ranging wildlife, and as a result, the impacts of many infectious agents on wildlife populations are unknown. Furthermore, coinfections—the simultaneous infection by multiple species of infectious agents in a host—also contribute to disease outcomes and further complicate the understanding of pathogen‐related effects, from individuals to populations (Telfer et al. 2010; Bordes and Morand 2011). Coinfections are considered the rule rather than the exception due to their ubiquity, yet most studies focus on single infectious species, which may lead to a biased perspective of wildlife health if effects are attributed to a single pathogen (Serrano and Millán 2014; Hoarau et al. 2020; Jones et al. 2023). When investigated, coinfections frequently are linked to reduced immune function and have more negative effects on the host compared to single pathogen infections (Jolles et al. 2008; Bordes and Morand 2011; Mabbott 2018; Shen et al. 2019; Hananeh et al. 2022). Pathogens that are immunosuppressive may thus increase a host's susceptibility to further infection.

Understanding coinfection dynamics requires conducting large scale diagnostic testing from presumably healthy animals. Popular game species, such as the wild turkey ( *Meleagris gallopavo* ), can provide opportunities to conduct large‐scale projects and investigate coinfections. Centuries of habitat destruction and overhunting resulted in historical lows in turkey abundance during the early 1900s (Earl et al. 1990; Dickson 1992). However, successful management efforts have led to dramatic increases in populations over the past several decades. Wild turkeys are currently widely distributed across North America at high densities varying by state and have a population size of approximately 5 million estimated throughout their range in the United States of America (USA) (Chamberlain et al. 2022). Despite the current wild turkey conservation status being listed as least concern (BirdLife International 2018), populations in recent years show potential declines, and ranges have been shown as stable or expanding. As a result, it is difficult to assess the wild turkey's conservation status, especially given the inconsistent monitoring methods among states (Chamberlain et al. 2022). Like many other wildlife species, wild turkeys harbor a variety of pathogens (Hafez and Shehata 2024) which may pose increased disease risk in higher population densities (Rifkin et al. 2012). Surveillance of wild turkeys has revealed the retrovirus lymphoproliferative disease virus (LPDV) to be one of the most prevalent pathogens (Thomas et al. 2015; MacDonald, Jardine, Bowman, et al. 2019; MacDonald et al. 2022; Goodwin et al. 2025). LPDV is oncogenic and can result in fatal lymphoid tumors, but cancer only arises in 3%–5% of infected turkeys (Allison et al. 2014; Niedringhaus et al. 2019; Goodwin et al. 2026). Lesions associated with LPDV commonly include skin lesions, splenomegaly, and emaciation, but results are biased towards birds that have died due to infections (Niedringhaus et al. 2019; Hafez and Shehata 2024). True mortality rates of lymphoproliferative disease are not well defined but can be high in studies examining sick birds (Allison et al. 2014); however, it appears that most wild turkeys infected with LPDV are asymptomatic (Allison et al. 2014; Thomas et al. 2015; MacDonald et al. 2022; Shea, Gonnerman, Blomberg, Sullivan, Milligan, and Kamath 2022).

LPDV has recently been linked to reduced clutch size in Maine wild turkeys, suggesting population‐level effects via pathological outcomes other than oncogenesis, such as immunosuppression (Shea 2021). Thus, the effects of LPDV on wild turkey populations may not be apparent. The same study in Maine revealed that another oncogenic retrovirus, reticuloendotheliosis virus (REV), was associated with reduced hen survival (Shea 2021). High LPDV and REV prevalences have also been observed in both healthy and sick turkeys in Pennsylvania (MacDonald et al. 2022; Adcock et al. 2024). Common lesions associated with REV are similar to those associated with LPDV, including splenomegaly and emaciation, but do not include skin lesions (Niedringhaus et al. 2019; Hafez and Shehata 2024). Despite these surveillance efforts, variation in retrovirus infection across the landscape remains unknown. Identifying the risk factors associated with higher infection can help inform future surveillance efforts, targeting more specific host groups, species, or locations, which can then be used to test future hypotheses (e.g., drivers of infection, mechanisms of transmission) (Paull et al. 2012). In addition, understanding higher‐risk groups may then lead to management strategies that reduce future outbreaks and slow transmission.

Additionally, if LPDV is immunosuppressive, there may be an association with parasites or another pathogen. However, it is unclear how frequently coinfections occur with retroviruses and other turkey pathogens (e.g., internal parasites, *Mycoplasma* bacteria) (Hoffman et al. 1997; Hafez and Shehata 2024). Therefore, the objectives of this study were to: (1) determine the prevalence of LPDV, REV, *Mycoplasma*, and internal parasites in wild turkeys across four Wildlife Management Units (WMUs) in Pennsylvania; (2) identify potential risk factors associated with these pathogens; and (3) estimate how coinfections are structured in turkeys and whether infections with retroviruses are related to infections with parasites. We predict there will be potential regional variation in infections based on the variable landscapes across Pennsylvania (Casalena 2018). Older turkeys may also be more commonly infected with retroviruses and parasites due to their longer exposure time. Finally, we expected turkeys infected with retroviruses to have higher parasite infections due to the potential effect of increased susceptibility to infection. To answer these questions, we employed active disease surveillance efforts from 2022 to 2025 across Pennsylvania landscapes as part of a broader wild turkey health study.

## 2 Materials and Methods

### 2.1 Study Design, Turkey Trapping, and Sampling

**FIGURE 1:**
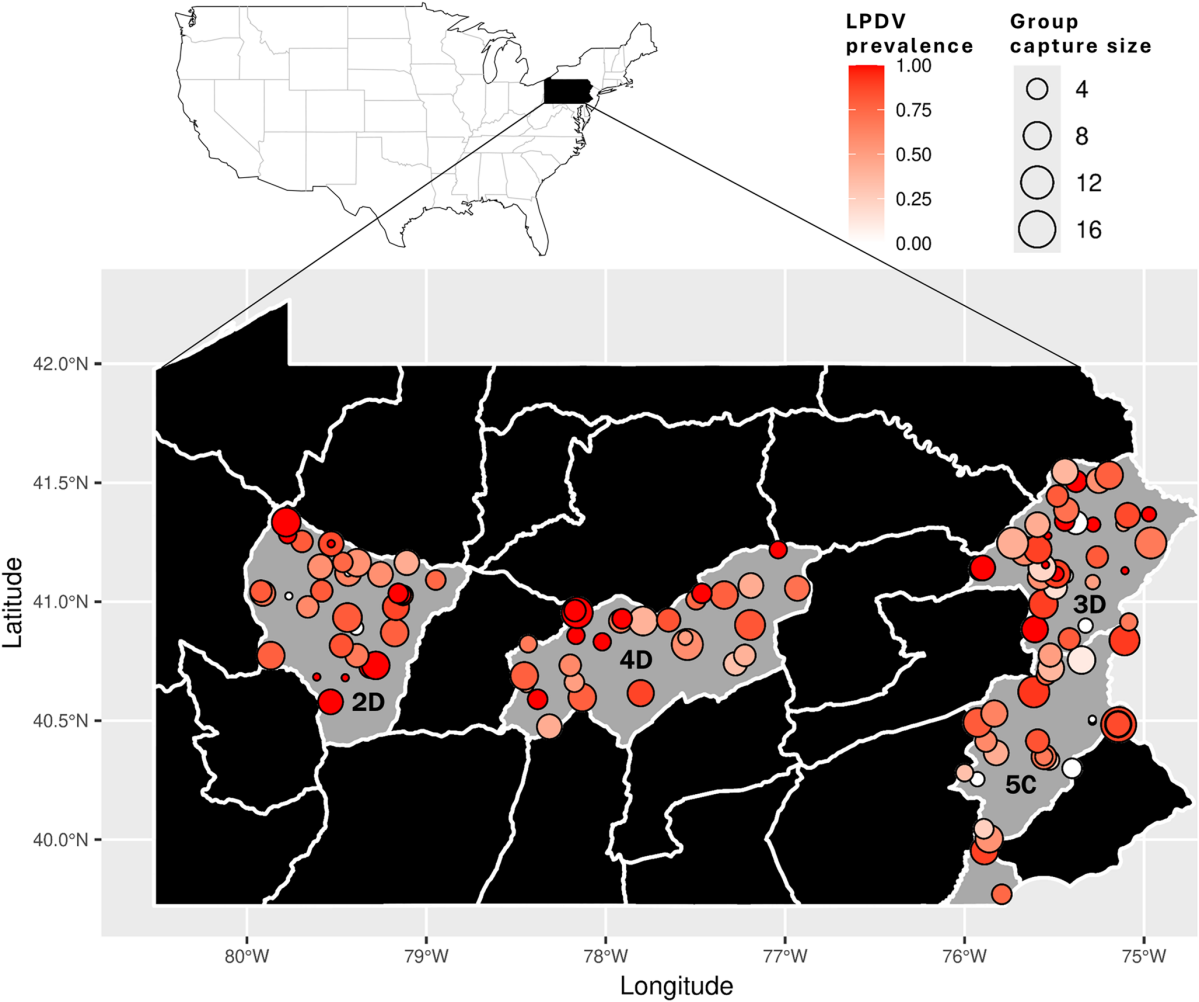
Map of United States (top) and Pennsylvania (bottom) showing wild turkey ( *Meleagris gallopavo* ) sampling sites (circles). White borders represent wildlife management unit (WMU) boundaries; the study areas included WMUs 2D, 3D, 4D, and 5C. Size of circles indicates group size of turkeys at time of capture. The red gradient represents lymphoproliferative disease virus (LPDV) prevalence, with darker red circles indicating higher LPDV prevalence and lighter red circles indicating lower LPDV prevalence.

Samples used in this study were part of a larger study led by the Pennsylvania Game Commission, in which turkey populations were monitored throughout Pennsylvania, USA for 4 years. Sampling locations were distributed throughout four of the 22 WMUs, which are geographic areas used to actively manage and monitor wild turkey populations and other species (Casalena 2018). Four WMUs (2D, 3D, 4D, 5C; Figure 1) were selected as study areas based on their variability in landscape features. Briefly, WMU 2D contains high mixed woodlands (deciduous/coniferous) and moderate agricultural and developed landscapes; 3D contains high mixed woodlands and low agricultural and developed landscapes; 4D contains moderate mixed woodlands and moderate agricultural and developed landscapes; and 5C contains low mixed woodlands and high agricultural and developed landscapes. Turkey harvest rates, survival, densities, and abundances were shown to vary across Pennsylvania WMUs prior to the study (Casalena 2018; Winter et al., forthcoming).

The Pennsylvania Game Commission trapped wild turkeys throughout 27 counties across these four WMUs during the months December–March from 2021 to 2025 accounting for four trapping seasons. Field crews trapped live turkeys at baited sites using rocket nets, following similar techniques to other studies (Kurzejeski et al. 1987; Eriksen et al. 1993; Delahunt et al. 2011). The number of turkeys trapped at sites (i.e., group capture size) was not always indicative of flock size as the size of the rocket net limited the number that could be captured. Thus, some captures had few birds netted despite a large flock. Each turkey was sexed, aged as juvenile or adult (Brenneman 1996), banded with a rivet‐style leg band (National Band and Tag Co., Louisville, Kentucky, USA), and fitted with a GPS backpack‐style transmitter (e‐obs digital telemetry, Munich, Germany). Data from GPS transmitters are not presented in this study. At each trapping site, landscape type was identified as agricultural (AG: a field with row crops), forested opening (FO: a small field surrounded by woods), or other (O: an area different than agricultural and forested opening). Venous whole blood samples (1.5–2 mL) were collected from the medial metatarsal, brachial, or jugular vein; 500 μL was placed in an anticoagulant (ethylenediaminetetraacetic acid; EDTA) blood collection tube, while the remaining blood was placed in a serum blood collection tube, allowed to clot up to an hour, centrifuged at 3500 RPM for 10 min, and the separated serum removed and placed in a cryovial. Tracheal/choanal samples were collected by inserting sterile synthetic swabs into the opening of the trachea and bringing the swab up through the choanal cleft, then placing the swab into a cryovial with 500 μL phosphate buffered saline (PBS). All samples were placed on ice packs or refrigerated for up to 24 h, stored in a −20°C freezer if held for more than 24 h, and then shipped on ice to the Wildlife Futures Program (New Bolton Center, University of Pennsylvania, Kennett Square, Pennsylvania). Whole blood was stored in a −20°C freezer until testing. Fecal samples were also collected opportunistically to facilitate parasitological sampling and stored in 10% buffered formalin at room temperature. Turkey capture and handling was completed under the Pennsylvania State University Institutional Animal Care and Use Committee (Protocol # PROTO202202180), while disease sample collection was performed by Pennsylvania Game Commission in compliance with review by their research committee and submitted for diagnostic testing.

### 2.2 Retrovirus Testing

DNA from whole blood samples was extracted using a DNeasy Blood and Tissue Kit (Qiagen, Valencia, California, USA) according to the manufacturer's protocol, except for the following modifications: started with 5 μL blood, incubated blood for 15 min, and used 100 μL buffer AE to elute. PCR protocols for LPDV and REV were performed according to Alger et al. (2015) and Davidson et al. (1995), respectively, with slight modifications made. For LPDV, reactions were performed in a 25 μL total volume: 12.5 μL Invitrogen Platinum II Hot‐Start PCR Master Mix 2X (Thermo Fisher Scientific, Waltham, Massachusetts, USA), 9 μL molecular grade water, 2.5 μL template DNA (range 7–178 ng/μL), and 0.5 μL of each primer (10 μM). LPDV PCR reactions were run on a ProFlex PCR System (Applied Biosystems, East Lyme, Connecticut, USA) under the following cycling conditions: 94°C for 2 min; followed by 35 cycles of 94°C for 30 s, 58°C for 30 s, and 68°C for 1 min; and a final extension at 68°C for 5 min. The primers for LPDV included the forward (ATGAGGACTTGTTAGATTGGTTAC) and reverse (TGATGGCGTCAGGGCTATTTG). For REV, 25 μL reactions were prepared as above, except that the template DNA was diluted 1:10. REV PCR reactions were run under the following cycling conditions: 94°C for 2 min; followed by 35 cycles of 94°C for 30 s, 60°C for 30 s, and 68°C for 1 min; and a final extension at 68°C for 5 min. The primers for REV included the forward (CATACTGGAGCCAATGGTT) and reverse (AATGTTGTACCGAAGTACT). For both LPDV and REV, negative controls (molecular‐grade water) and positive controls (synthetic DNA fragments, gene blocks; Thermo Fisher Scientific, Waltham, Massachusetts, USA) were prepared for each run, and PCR success was determined by visualizing PCR products on an E‐Gel EX 1% agarose gel with an E‐Gel Power Snap Electrophoresis System (Thermo Fisher Scientific, Waltham, Massachusetts, USA). Any samples presenting faint bands were rerun using the same parameters, and resulting bright bands were deemed positive. A subset of these discrepant samples was sent to Eurofins Genomics (Louisville, Kentucky, USA) for direct Sanger sequencing, which confirmed sample positivity.

### 2.3 Parasitology Testing

Fecal samples were assessed for internal parasites at the Penn Vet Clinical Parasitology Diagnostic Laboratory (University of Pennsylvania, Philadelphia, Pennsylvania). Double centrifugal fecal flotation procedure using Sheather's sugar flotation solution (JorVet, Loveland, Colorado, USA) and morphological identification of the diagnostic stages of all internal endoparasites from feces were performed as recommended in Zajac et al. (2021). Briefly, an aliquot of 1–3 g of feces was thoroughly mixed with approximately 12 mL of tap water in a plastic cup. The resulting suspension was strained and transferred to a 15 mL conical centrifuge tube. Tubes were centrifuged at 2500 RPM for 10 min, after which the supernatant was discarded and the sediment resuspended in approximately 15 mL of the sugar flotation solution (specific gravity 1.27). Centrifugation was repeated as described above. The tube was filled with sugar flotation solution to form a reverse meniscus, and a coverslip was placed on top. After 10 min, the coverslip was transferred to a glass slide and examined systematically at 10× objective. Parasites were identified based on morphology via light microscopy to the lowest taxonomic level when possible, and hosts were deemed infected or non‐infected with a specific parasite taxon.

### 2.4 Mycoplasma Testing

To test for *Mycoplasma* spp., tracheal/choanal swabs were submitted to the Pennsylvania Animal Diagnostic Laboratory System New Bolton Center Molecular Laboratory, and samples were stored at −80°C until tested. Each swab was resuspended in phosphate‐buffered saline (PBS; Thermo Fisher Scientific, Waltham, Massachusetts, USA), and DNA was extracted using the MagMax Core Nucleic Acid Purification kit (Thermo Fisher Scientific, Waltham, Massachusetts, USA) following the manufacturer's protocol. Due to very few positives, only sub‐samples from 2022 to 2024 were selected for testing. Samples were tested for *Mycoplasma synoviae* and *Mycoplasma gallisepticum* using the IDEXX RealPCR MG/MS Multiplex DNA Mix (IDEXX, Scarborough, Maine, USA) according to the manufacturer's instructions. Real‐time PCR for *Mycoplasma meleagridis* was conducted using previously published primers and probe set (Raviv and Kleven 2009) with the following modification: PCR reactions were carried out in a 25 μL reaction volume consisting of 12 μL VetMAX qPCR Master Mix (Thermo Fisher Scientific, Waltham, Massachusetts, USA), 0.7 μL of each primer (10 μM), 1 μL of probe (10 μM), 1 μL of internal control assay (synthetic DNA; Thermo Fisher Scientific, Waltham, Massachusetts, USA), 5 μL of the DNA template, and 4.6 μL of water. Amplification was performed on either a 7500 or QuantStudio 5 Real‐Time PCR System (Thermo Fisher Scientific, Waltham, Massachusetts, USA). The primers for *M. meleagridis* included the forward (AACAAGGTATCCCTACGAGAAC) and reverse (CTCAGAGCCTTAAACCAAGTCA), as well as the probe (CCTCCTTTCTACGGAGTACATTAGTT). The cycling conditions were as follows: 95°C for 10 min, followed by 40 cycles of 95°C for 15 s and 60°C for 30 s.

### 2.5 Statistical Analyses

Prevalence for each pathogen or parasite was defined as the number of infected hosts divided by the number of hosts sampled (Bush et al. 1997). Coinfection prevalence was calculated similarly and defined as the number of coinfected hosts divided by only those hosts tested for both pathogens. 95% confidence intervals were computed according to Sokal and Rohlf (1995) for sampling efforts in each study area. A linear regression model was fit to determine the relationship between LPDV prevalence (predictor variable) and group capture size (response variable) among all capture sites. Group capture size was not included in additional models if not significant. A mixed effects logistic regression model was fit to test for the effects of age, sex, year, study area, and landscape type (predictor variables) on LPDV infection (response variable), treating capture event as a random effect due to the potential for LPDV to be transmitted within groups of turkeys. Due to the low number of infections, models were not fit for REV and *Mycoplasma* spp. infections (see Results). The number of different parasite species (i.e., species richness) was summed for individual turkeys to use as a metric of parasite diversity (i.e., the total number of parasite species per host). A Poisson regression model was then fit to test the effects of LPDV, age, sex, year, study area, and landscape type (predictor variables) on parasite species richness (response variable). Additionally, individual logistic regression models were fit for each parasite species (prevalence > 5%) and coinfection with LPDV and parasites to test the same predictors on parasite infection. For the above models, the “Anova” function was used in the “car” package version 3.1–3 (Fox and Weisberg 2019) to determine global *p*‐values for each variable, residuals were visualized to assess normality of data, and multicollinearity among predictor variables was checked with the Variance Inflation Factor (VIF = 1, no correlation, VIF = 2–4 moderate correlation, VIF = 5 high correlation). Any predictor variables with a VIF ≥ 5 were excluded from models. For any significant factors in models with three or more levels, a pairwise comparison test was performed with the Tukey correction. The model interaction term of sex:age was initially included in all models but removed when not significant (*p* ≥ 0.05). A co‐occurrence analysis was performed using the “cooccur” package version 1.3 (Griffith et al. 2016) to determine whether species pairs co‐occur at a frequency less than random (negative associations), at random (no association), or more than random (positive associations). Only pathogens with a prevalence > 5% were analyzed in the co‐occurrence analysis to improve statistical power. A linear regression model was also fit to determine the relationship between LPDV prevalence (predictor variable) and parasite prevalence (response variable) among sampled counties with at least two samples per county to determine any county‐level effects. All analyses were conducted in R version 4.4.1 (R Core Team 2020). A list of all models and their associated variables is presented in Table S1.

## 3 Results

### 3.1 LPDV, REV, and Mycoplasma

**FIGURE 2:**
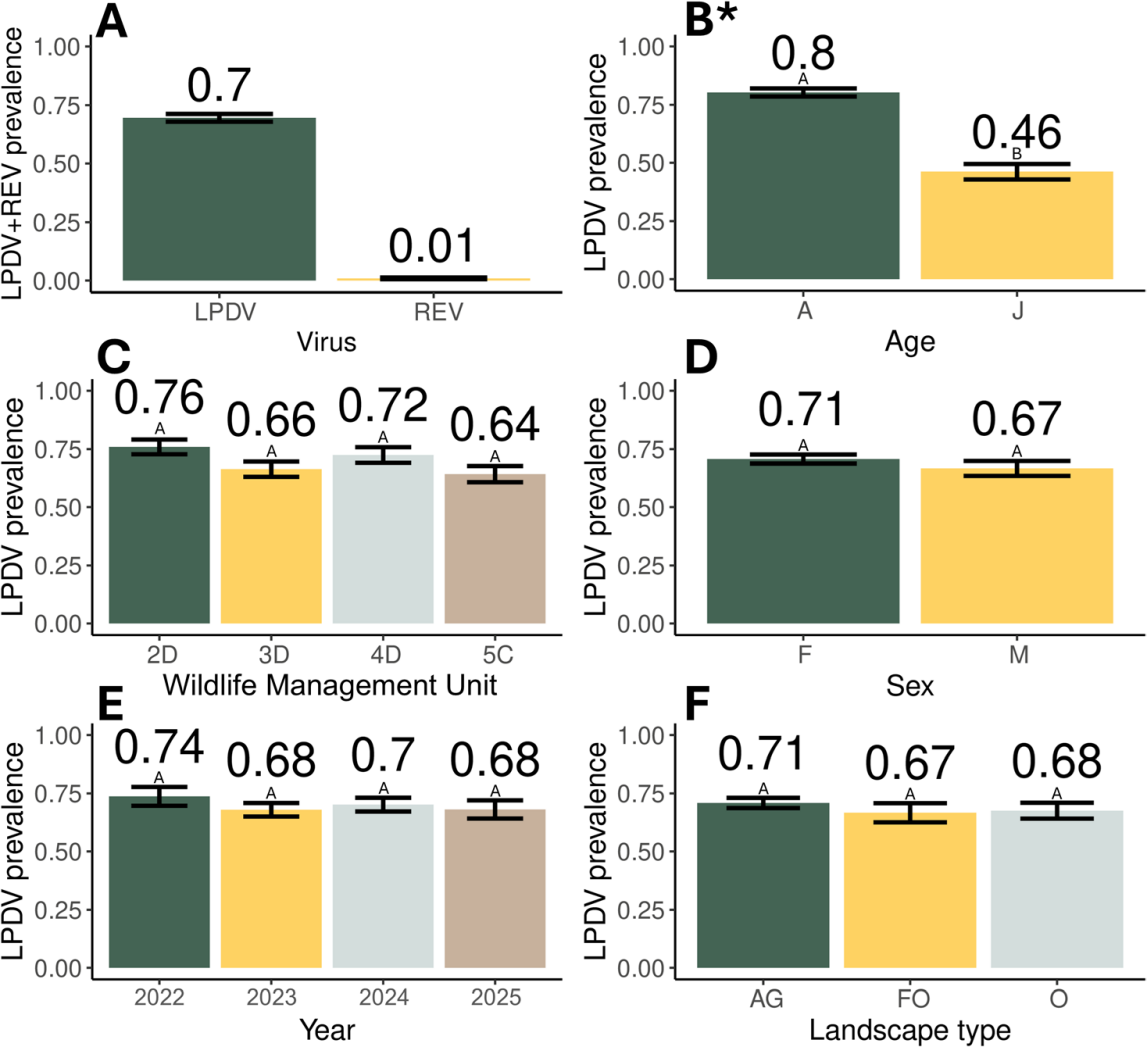
Virus infection in a sample (*n* = 756) of Pennsylvania wild turkeys ( *Meleagris gallopavo* ) showing differences between (A) lymphoproliferative disease virus (LPDV) and reticuloendotheliosis virus (REV), as well as LPDV prevalence with (B) age, (C) Wildlife Management Unit, (D) sex, (E) year, and (F) landscape type (AG = agricultural, FO = forested opening, and O = other). An * indicates variable significance in the model (*p* < 0.05), different letters between bars indicate significant pairwise differences, and error bars indicate standard error.

A total of 756 turkeys were tested for both viruses and included the years 2022 (*n* = 118), 2023 (*n* = 259), 2024 (*n* = 238), and 2025 (*n* = 141), and the WMUs 2D (*n* = 183), 3D (*n* = 205), 4D (*n* = 178), and 5C (*n* = 190). LPDV and REV prevalence in the entire study region was 70% (526/756) and 1% (7/756) (Figure 2A), respectively, and ranged from 64% to 76% for LPDV and 0%–2% for REV among the four WMU study areas (Table 1). LPDV was detected in all 27 counties sampled (27/27) and REV was detected in 19% (5/27). Among all 141 turkey capture sites, LPDV prevalence and group capture size varied throughout the entire study region (Figure 1). There was no relationship between LPDV prevalence and group capture size among capture sites (*R* ^2^ = 0.01, *r* = 0.04, *F*_1,139_ = 0.35, *p* = 0.56). A total of 746 individuals remained in the model after accounting for missing metadata. In the LPDV mixed effects logistic regression model, adult turkeys were significantly more likely to be infected with LPDV than juveniles (odds ratio = 6.0; 95% CI = 4.0–9.2, *p* < 0.001) (Figure 2B). Study area (*p* = 0.54) (Figure 2C), sex (*p* = 0.66) (Figure 2D), year (*p* = 0.48) (Figure 2E), and landscape type (*p* = 0.77) (Figure 2F) were not related to LPDV infection. The prevalence of *M. gallisepticum* , *M. meleagridis* , and *M. synoviae* was 0% (0/513), 4% (11/289), and 2% (9/513), respectively (Table 2).

**TABLE 1:** Infection prevalence and 95% confidence intervals (CI) of lymphoproliferative disease virus (LPDV), reticuloendotheliosis virus (REV), and internal parasites from Pennsylvania wild turkeys ( *Meleagris gallopavo* ) by Wildlife Management Unit.

Wildlife management unit	LPDV prevalence (no. infected/no. examined)	LPDV 95% CI (%)	REV prevalence (no. infected/no. examined)	REV 95% CI (%)	Parasite prevalence (no. infected/no. examined)	Parasite 95% CI (%)
2D	76% (139/183)	(69–82)	0% (0/183)	(0–2.0)	55% (65/118)	(46–64)
3D	66% (136/205)	(59–73)	1% (1/205)	(0–3.0)	79% (117/149)	(71–85)
4D	72% (129/178)	(65–79)	2% (3/178)	(0.4–4.9)	63% (48/76)	(51–74)
5C	64% (122/190)	(57–71)	2% (3/190)	(0.3–4.5)	56% (101/179)	(49–64)
Total	70% (526/756)	(66–73)	1% (7/756)	(0.4–2)	63% (331/522)	(59–68)

**TABLE 2:** Infection and coinfection prevalence among all parasites and pathogens from Pennsylvania wild turkeys (*Meleagris gallopavo*). Coinfection prevalence represents the proportion of coinfected individuals among only those individuals sampled for both parasites and/or pathogens.

Pathogen/parasite	Infected	Samples (*n*)	Prevalence (%)
LPDV	526	756	70
REV	7	756	1
PARA	347	545	64
MG	0	513	0
MM	11	289	4
MS	9	513	2
LPDV+REV^a^	7	756	1
LPDV+PARA	214	522	41
MS+MM^b^	0	223	0
MS+LPDV	3	371	1
MS+PARA	3	371	1
MM+LPDV	7	223	3
MM+PARA	5	223	2
MS+LPDV+PARA	2	371	1
MM+LPDV+PARA	5	223	2

Abbreviations: LPDV, lymphoproliferative disease virus; MG, *Mycoplasma gallisepticum* ; MM, *Mycoplasma meleagridis* ; MS, *Mycoplasma synoviae* ; PARA, internal parasites; REV, reticuloendotheliosis virus.

^a^ All REV‐positive samples were also LPDV‐positive, thus REV associations are not shown again.

^b^ Due to no MS+MM coinfections, this association is not shown again.

### 3.2 Parasites

**FIGURE 3:**
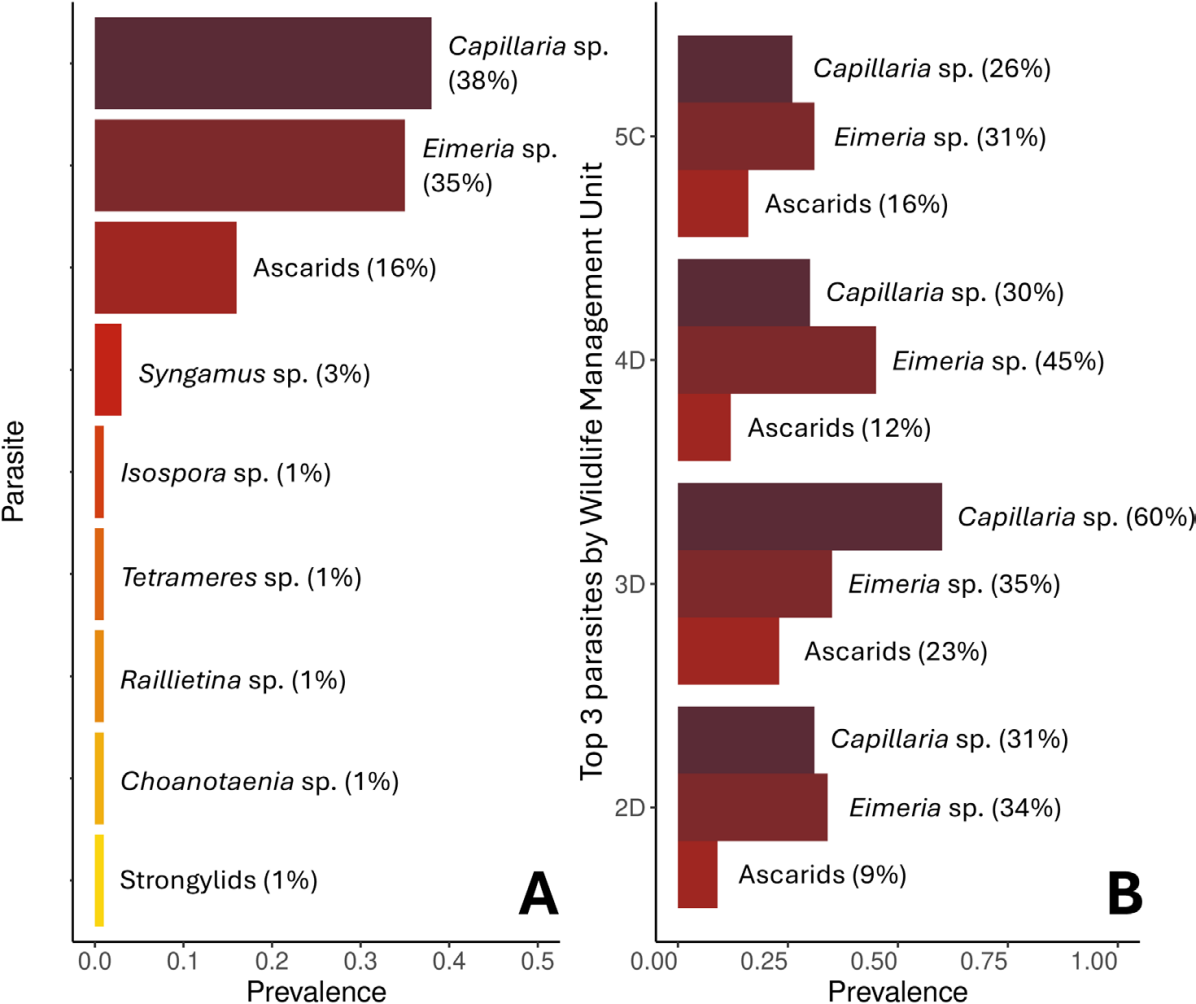
(A) Prevalence (*n* = 513) of all internal parasites recovered from Pennsylvania wild turkeys ( *Meleagris gallopavo* ) and (B) prevalence of the top three most common parasites by Wildlife Management Unit.

A total of 545 turkeys were tested for parasites, but only 522 contained metadata. Among these 522 samples, they included the years 2022 (*n* = 51), 2023 (*n* = 176), 2024 (*n* = 195), and 2025 (*n* = 100) and the WMUs 2D (*n* = 118), 3D (*n* = 149), 4D (*n* = 76), and 5C (*n* = 179). Parasites were recovered from 63% (331/522) of captured turkeys, with prevalence ranging from 55% to 79% across WMUs (Table 1). Ninety‐six percent (26/27) of the counties in the entire study region included at least one turkey with at least one parasite species. A total of nine unique parasite taxa were identified to the order or genus level and included two cestodes (Phylum: Platyhelminthes), two coccidia (Phylum: Apicomplexa), and five nematodes (Phylum: Nematoda). Prevalence of each parasite taxon ranged from 1% to 38% (Figure 3A). *Capillaria* sp., *Eimeria* sp., and ascarid nematodes (order: Ascaridida) represented the most prevalent parasites (16%–38%), while all other parasites had a prevalence of 3% or lower. Among the top three most prevalent parasite taxa, parasite prevalence was structured similarly among three of the four WMUs (2D, 4D, 5C), where *Eimeria* sp. was the most prevalent taxon, and ascarid nematodes were the least prevalent (Figure 3B). In WMU 3D, *Capillaria* sp. was the most prevalent taxon, while ascarid nematodes were the least prevalent.

**FIGURE 4:**
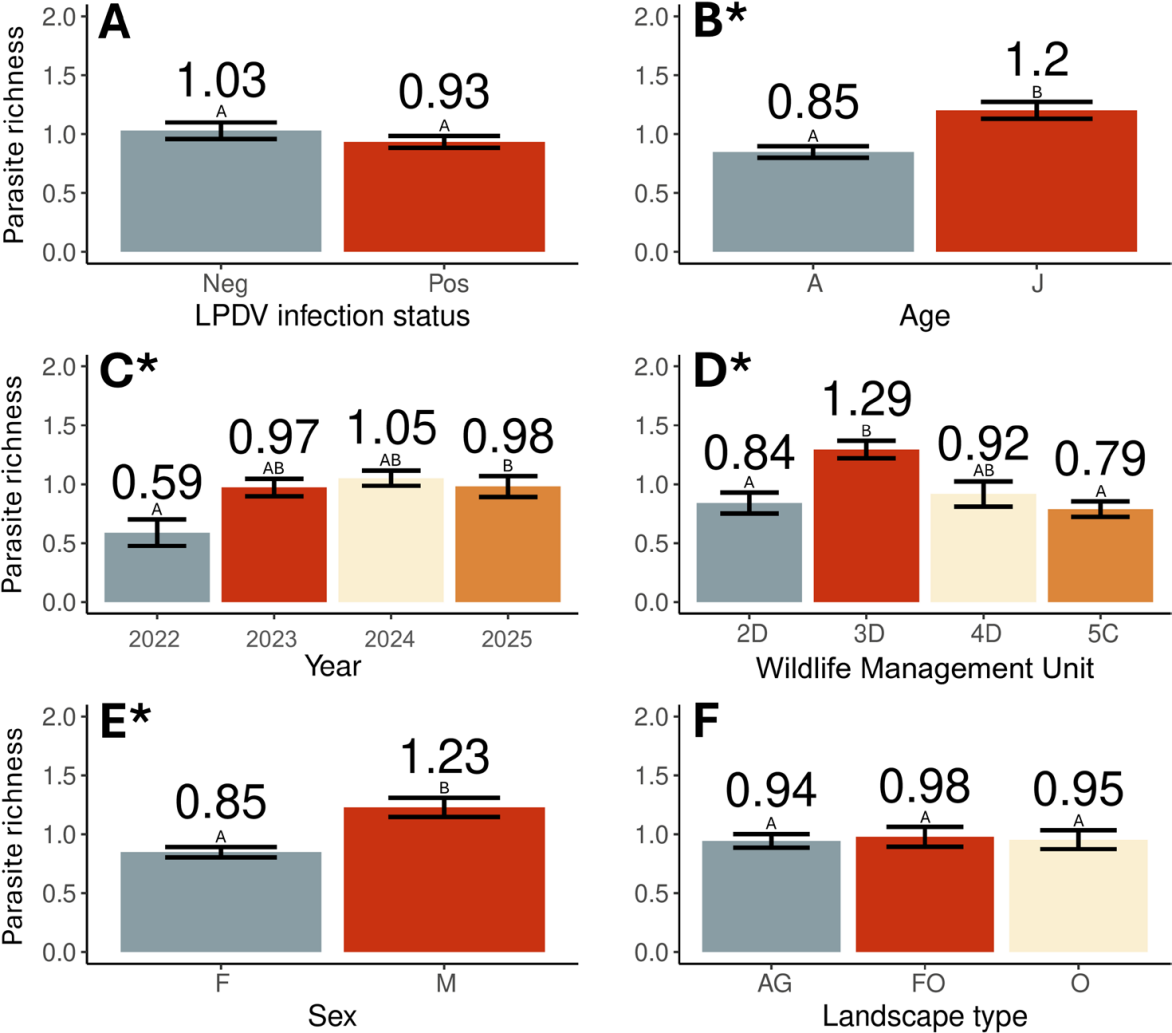
Average parasite species richness in a sample (*n* = 513) of Pennsylvania wild turkeys ( *Meleagris gallopavo* ), showing differences in richness by (A) lymphoproliferative disease virus (LPDV) infection status, (B) age, (C) year, (D) Wildlife Management Unit, (E) sex, and (F) landscape type (AG = agricultural, FO = forested opening, and O = other). An * indicates variable significance in the model (*p* < 0.05), different letters between bars indicate significant pairwise differences, and error bars indicate standard error.

For the parasite models, a total of 513 samples remained after accounting for missing metadata and overlapping virus data. Parasite species richness did not vary with LPDV infection (χ^2^_1_ = 0.5, *p* = 0.46) (Figure 4A) or landscape type (χ^2^_2_ = 0.5, *p* = 0.79) (Figure 4F), whereas there were significant effects of age (χ^2^_1_ = 12.4, *p* < 0.001) (Figure 4B), year (χ^2^_3_ = 8.8, *p* = 0.03) (Figure 4C), study area (χ^2^_3_ = 22.3, *p* < 0.001) (Figure 4D), and sex (χ^2^_1_ = 12.6, *p* < 0.001) (Figure 4E) on parasite species richness. Specifically, parasite species richness was higher in juveniles (*z* = 3.56, *p* < 0.001), the year 2025 (*z* = 2.8, *p* = 0.02) only when compared to 2022, WMU 3D only when compared to 2D (*z* = 3.4, *p* = 0.004) and 5C (*z* = 4.4, *p* < 0.001), and males (*z* = 3.6, *p* < 0.001).

The *Capillaria* sp. model indicated significant effects of age (*p* < 0.001), sex (*p* < 0.001), year (*p* = 0.03), and study area (*p* < 0.001) on *Capillaria* sp. infection. Specifically, infection was higher in juveniles (odds ratio = 2.7; 95% CI = 1.7–4.3, *p* < 0.001), males (odds ratio = 2.2; 95% CI = 1.4–3.6, *p* < 0.001), the year 2025 (odds ratio = 3.8; 95% CI = 1.5–10.3, *p* < 0.001) only when compared to 2022, and WMU 3D compared to 2D (odds ratio = 4.2; 95% CI = 2.4–7.4, *p* < 0.001), 4D (odds ratio = 4.7; 95% CI = 2.4–9.4, *p* < 0.001), and 5C (odds ratio = 5.5; 95% CI = 3.3–9.3, *p* < 0.001). *Capillaria* sp. infection did not vary with LPDV infection (*p* = 0.92) or landscape type (*p* = 0.61).

The *Eimeria* sp. model indicated a significant effect of landscape type (*p* = 0.04) on *Eimeria* sp. infection, with infection slightly higher in the “forested opening” category (odds ratio = 2.1; 95% CI = 1.2–3.6, *p* = 0.04) only when compared to the “other” category. *Eimeria* sp. infection did not vary with LPDV infection (*p* = 0.12), age (*p* = 0.59), sex (*p* = 0.13), study area (*p* = 0.55), or year (*p* = 0.09).

Finally, the ascarid model indicated significant effects of age (*p* = 0.004) and study area (*p* = 0.03) on ascarid infection, with infection higher in juveniles (odds ratio = 2.2; 95% CI = 1.3–3.9, *p* = 0.004) and WMU 3D (odds ratio = 3.0; 95% CI = 1.4–6.8, *p* = 0.03) only when compared to 2D. Ascarid infection did not vary with LPDV infection (*p* = 0.64), sex (*p* = 0.11), year (*p* = 0.91), or landscape type (*p* = 0.41).

### 3.3 Coinfections

**FIGURE 5:**
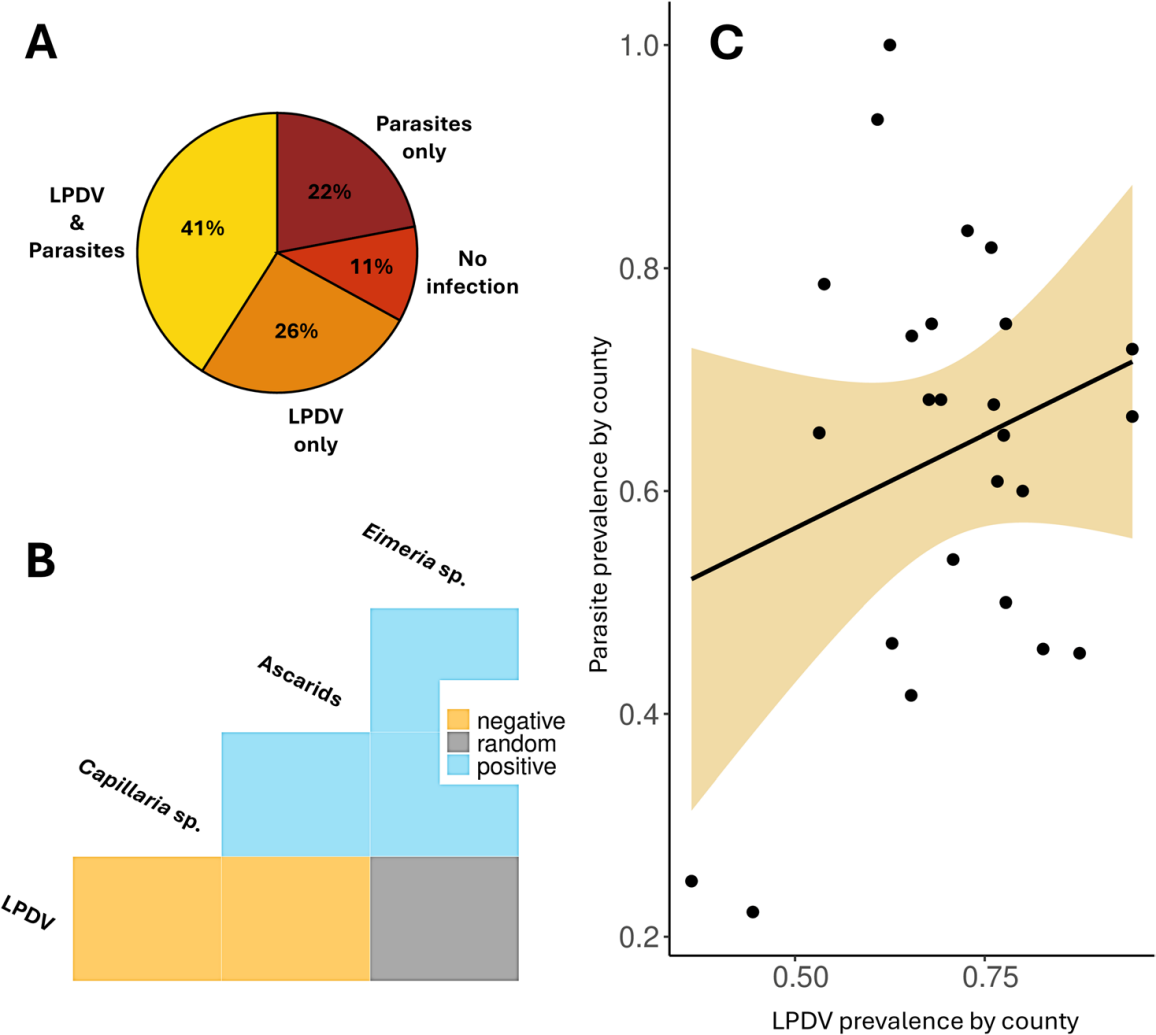
(A) Proportions of lymphoproliferative disease virus (LPDV) and parasite coinfections in a sample (*n* = 522) of Pennsylvania wild turkeys ( *Meleagris gallopavo* ). (B) Cooccurrence matrix showing relationships between the most prevalent pathogens among individual turkeys. (C) Scatterplot showing the relationship between parasite prevalence and LPDV prevalence within the Pennsylvania counties encompassing the entire study region.

All seven REV‐positive turkeys were also positive for LPDV, and thus 1% (7/756) of the captured turkeys were coinfected with LPDV and REV. Among the turkeys tested for both parasites and viruses, 41% (214/522) were coinfected with both LPDV and parasites, whereas 26% (135/522) were infected with only LPDV, 22% (114/522) were infected with only parasites, and 11% (59/522) were not infected with either parasites or viruses (Figure 5A). All other coinfection associations between viruses, parasites, and *Mycoplasma* spp. were low, ranging from 0% to 3% (Table 2). The coinfection model indicated significant effects of age (*p* = 0.002) and year (*p* = 0.02) on coinfection, with coinfection higher in adults (odds ratio = 1.9; 95% CI = 1.3–2.9, *p* = 0.002) and 2024 (odds ratio = 2.8; 95% CI = 1.4–6.3, *p* = 0.04) only when compared to 2022. Coinfection did not vary with sex (*p* = 0.52), study area (*p* = 0.3), or landscape type (*p* = 0.29). The co‐occurrence analysis (Figure 5B) indicated positive associations between *Capillaria* sp. and *Eimeria* sp. (*p* = 0.007), *Eimeria* sp. and ascarids (*p* = 0.01), and *Capillaria* sp. and ascarids (*p* < 0.001); negative associations between LPDV and *Capillaria* sp. (*p* = 0.04) and LPDV and ascarids (*p* = 0.02); and no association between LPDV and *Eimeria* sp. (*p* = 0.07). Among the Pennsylvania counties encompassing the entire study region, there was no relationship between LPDV and parasite prevalence (*R* ^2^ = 0.02, *r* = 0.25, *F*_1,23_ = 1.49, *p* = 0.23) (Figure 5C).

## 4 Discussion

### 4.1 Retroviruses

This study sheds light on some of the biological and ecological factors associated with LPDV and parasite infections in wild turkeys from Pennsylvania, highlighting how these infectious agents are distributed across Pennsylvania and within turkey populations. Results from this study indicate that LPDV has persisted at consistently high levels over time, in line with previous surveillance efforts in Pennsylvania (Allison et al. 2014; MacDonald et al. 2022; Adcock et al. 2024), suggesting that the virus is likely enzootic in the state. In other countries and states in the USA, LPDV prevalences vary by state (26%–83%) but tend to be higher in the northeast (75%; Thomas et al. 2015) and Ontario, Canada (65%; MacDonald, Jardine, Bowman, et al. 2019) compared to lower prevalences in the southeast (48%) and central (23%) USA (Thomas et al. 2015). REV prevalences are generally low in studies with live‐captured birds (4%–16%; MacDonald, Jardine, Bowman, et al. 2019; Cox et al. 2022; Shea, Gonnerman, Blomberg, Sullivan, Milligan, and Kamath 2022). However, REV prevalence tends to be higher from turkeys collected as dead and clinically ill, as well as when other tissues are tested, such as spleen, liver, and bone marrow (44%; Adcock et al. 2024), highlighting the potential pathogenicity of REV. Lastly, parasite species richness was consistent across years but notably lower in 2022 compared to 2025, though this was likely due to less sampling in 2022 (*n* = 51) compared to 2023–2025 (*n* ≥ 100/year).

### 4.2 Parasites

Parasite data presented in this study capture the minimum diversity of internal parasites from Pennsylvania wild turkeys, serving as a useful baseline reference for future studies. Among the nine parasite taxa detected in this study, *Capillaria* sp., *Eimeria* sp., ascarids (including *Ascaridia* sp. and *Heterakis* sp.), *Syngamus* sp. (including *Syngamus trachea*), and *Raillietina* sp. have all been previously reported in Pennsylvania wild turkeys (Williams 1931; Kozicky 1948; Schorger 1966; Davidson and Wentworth 1992; Latham 2017; Greenawalt et al. 2020; MacDonald et al. 2022). However, to the best of our knowledge, *Isospora* sp., *Tetrameres* sp., *Choanotaenia* sp. (including *Choanotaenia infundibulum*) and strongylids (including *Trichostrongylus* and *Amidostomum* sp.) have not been reported. This may be due to the limited number of parasite surveys in Pennsylvania and northeastern USA wild turkey populations. We acknowledge that these findings are only reflective of the parasite eggs and oocysts shed in feces at the time of capture, which can vary temporally and under different environmental and host conditions (Reece et al. 2017). Notably, turkeys from this study were not euthanized to obtain parasite data, emphasizing the practicality of using non‐invasive methods to assess parasite diversity. Two nematodes (*Capillaria* sp., 38%; ascarids, 16%) and one coccidium (*Eimeria* sp., 35%) were the most prevalent and had similar distributions in three of the four WMUs (Figure 3B). The higher prevalence of *Capillaria* sp. in WMU 3D could be due to greater regional abundances of earthworms, which act as hosts in the life cycles of some species of *Capillaria* (Cox 1968; Hafez and Shehata 2024). Depending on the species, the top three most common parasites found in this study are potentially pathogenic in wild and domestic turkeys (Dickson 1992; Hafez and Shehata 2024) and may be relevant when investigating turkey health. For example, some common parasites in turkeys (wild and domestic) are *Ascaridia dissimilis*, *Ascaridia galli* , *Heterakis gallinarum*, *Capillaria contorta*, *Eimeria adenoeides*, *Eimeria gallopavonis*, *Eimeria meleagrimitis*, *Eimeria meleagridis*, and *Eimeria dispersa*, all of which can cause emaciation and reduced feeding, with extent of disease increasing with severity of infection (Neimanis and Leighton 2004; Hafez and Shehata 2024).

### 4.3 Coinfections

Characterizing coinfection prevalence can further inform our understanding of how simultaneous infections arise and if they are more commonly associated with specific risk factors. Notably, all REV‐positive turkeys were also positive for LPDV. However, the coinfection prevalence among these two viruses was low (1%) due to the low number of REV‐positive turkeys. This LPDV‐REV coinfection prevalence falls in the general range from previous studies of wild turkeys in Maine (10%), Texas (0.5%), and Ontario, Canada (4%) (MacDonald, Jardine, Bowman, et al. 2019; Cox et al. 2022; Shea, Gonnerman, Blomberg, Sullivan, Milligan, and Kamath 2022). Among all other coinfections, the high pattern of infections with LPDV and parasites (41%) can be attributed to the overall high prevalence of LPDV (70%) and parasites (63%). Few studies have simultaneously examined wild turkeys for both retroviruses and internal parasites, thus making it difficult to make direct comparisons. One study in Mississippi noted various external and internal parasite species were associated with turkeys infected with LPDV, although coinfection rates were not reported (Thiemann et al. 2022). Another study in Maine found that LPDV‐infected turkeys simultaneously infected with the bacterial pathogens *M. gallisepticum* and *Salmonella pullorum* had varying coinfection rates of 51% and 2.6%, respectively (Shea, Gonnerman, Blomberg, Sullivan, Milligan, and Kamath 2022). Similar to Shea, Gonnerman, Blomberg, Sullivan, Milligan, and Kamath (2022), infections with avian *Mycoplasma* species can be high in other wild turkey populations, which may be due to variation in detection methods and/or detection of non‐pathogenic *Mycoplasma* spp. (Hoffman et al. 1997; MacDonald, Jardine, Rejman, et al. 2019). However, our low number of *Mycoplasma*‐infected turkeys is likely a result of both geographic‐induced variation and differences in prevalence between *Mycoplasma* spp. (Luttrell et al. 1992). The impact of coinfections on wild turkeys remains poorly understood, especially in the context of population management. Ongoing efforts are currently investigating the population‐level effects of these parasites, viruses, and coinfections on turkey reproduction (Smelter et al., forthcoming).

### 4.4 Regional Trends

We observed different regional trends between viruses and parasites. LPDV prevalence did not appear to vary between study areas, while parasite richness did. Landscape factors are known to influence the prevalence and distribution of parasites and pathogens. For example, gastrointestinal nematodes with free‐living infective stages are influenced by microclimate (e.g., temperature, humidity, soil type), which can influence parasite survival and prevalence (McSorley 2003; McCallum 2008). Different landscape features have been linked to LPDV infection in wild turkeys, such that LPDV prevalence was greater in forested areas in Maine but greater in agricultural areas in New York (Alger et al. 2017; Shea, Gonnerman, Blomberg, Sullivan, Milligan, and Kamath 2022). However, we did not find differences between landscape types in Pennsylvania, possibly suggesting that our landscape variables were too coarse a scale and more specific landscape features may be driving these associations. In the future, it would be informative to determine the environmental variables potentially driving the variation in the observed parasites and pathogens across study areas and years. Host population dynamics can also influence pathogen prevalence. Because experimental evidence suggests that LPDV is transmitted via close contact in domestic turkeys (McDougall et al. 1978), higher turkey densities may be driving LPDV prevalence, especially given that LPDV occurs throughout the entire eastern wild turkey distribution and varies greatly by state (Thomas et al. 2015; Chamberlain et al. 2022). However, previous work in Maine has shown that LPDV infection does not vary with flock size (Shea, Gonnerman, Blomberg, Sullivan, Milligan, and Kamath 2022), indicating that turkey densities alone may not explain LPDV prevalence. Other types of transmission, such as blood‐feeding insect vectors or specific behaviors (e.g., mating) are potential routes for avian retroviruses. For example, REV has been detected in cloacal swabs, eggs, semen, and insects such as mosquitoes (Hafez and Shehata 2024), while LPDV has been detected from cloacal and choanal swabs (Shea, Gonnerman, Blomberg, Sullivan, and Kamath 2022; Goodwin et al. 2024). To better understand the exact mechanisms of LPDV and REV transmission in wild turkeys, further experimental studies are required, as well as studies to determine if there is a relationship between mosquito densities and retrovirus prevalence.

### 4.5 Individual Trends

At the individual level, overall parasite species richness did not differ with LPDV infection, regardless of LPDV‐parasite coinfections being the most common association. This suggests that LPDV does not increase susceptibility to infection with internal parasites. This may be partly due to turkeys not having reduced immune function, although this was not part of this study. However, there were significant negative associations with LPDV and nematodes which could be explained by age‐related susceptibility, as LPDV prevalence was higher in adults, and *Capillaria* sp. and ascarid prevalences were higher in juveniles. Nematode parasites like *Capillaria* sp. and two turkey ascarids, *Ascaridia* sp. and *Heterakis* sp., can be transmitted via ingestion of eggs in soil or via an arthropod paratenic host (Augustine and Lund 1974; Hafez and Shehata 2024). Because young poults feed predominantly on animal matter, including a variety of soil‐dwelling arthropods (Dickson 1992), differences in feeding patterns may result in a higher probability of ingesting various species of nematodes earlier in life. In contrast, a chronic pathogen like LPDV, which is transmitted via close contact (McDougall et al. 1978), would be expected to have higher prevalence in adults due to their cumulative exposure over time. These findings are consistent with previous work showing LPDV is more commonly found in adult wild turkeys (Alger et al. 2017; Cox et al. 2022; Shea, Gonnerman, Blomberg, Sullivan, Milligan, and Kamath 2022; Thiemann et al. 2022). Alternatively, the higher prevalence observed in older turkeys may be due to increased mortality among young poults with underdeveloped immune systems when infected with the virus, thus are less likely to be captured (Niedringhaus et al. 2019). Finally, ascarid nematodes, *Capillaria* sp., and *Eimeria* sp. were all positively associated with each other and may have to do with the similarities in transmission strategies between parasite types (i.e., ingestion of eggs and oocysts in soil or via arthropods while foraging; Augustine and Lund 1974; Dickson 1992; Hafez and Shehata 2024). Overall, these results suggest that host demographics and behaviors are important in structuring coinfections with different pathogens, and LPDV and internal parasites are transmitted independently.

### 4.6 Study Limitations

Although this study addressed the main objectives, we acknowledge several limitations to the study methodology and associated results. For example, our coprology testing utilized a fecal flotation method (Sheather's sugar flotation solution) with a specific gravity of 1.27, which has advantages in detecting many common low‐density eggs and cysts found in turkeys, including nematodes and protozoans (Zajac et al. 2021). However, this method can limit the detection of denser parasite stages, such as trematode and cestode eggs. Thus, the reported results within may primarily reflect nematodes and coccidia. Additionally, this study sampled turkeys only during the colder winter months. This was mainly selected as a technique to facilitate attracting turkeys to baited sites when resources are more limited in the winter and preventing capture myopathy. We acknowledge that sampling only during a single season can lead to biases in the results presented, such that temporal trends in pathogen data will not be included in the analyzes, leading to omission of these data from future studies. We hope readers take these considerations with caution when interpreting our results.

## 5 Conclusions

Understanding the impacts of diseases on wildlife populations is challenging. However, conducting extensive surveillance and identifying risk factors are critical first steps in doing so. This study revealed that LPDV and internal parasites vary in how they are structured in turkeys across Pennsylvania, with different parasites and pathogen groups displaying unique infection dynamics representative of the biology of each species. The high coinfection rates found in wild turkeys may not be surprising, as wildlife species tend to harbor numerous pathogens. From a management perspective, these data present an exciting opportunity for use in wild turkey models, such that they can be linked to survival and reproductive outcomes that may help explain spatio‐temporal fluctuations in population dynamics. Given that turkeys collected in this study were alive and did not show overt signs of infection with any of the pathogens of interest, further investigation should focus on determining the sublethal effects of these parasites and pathogens on turkey reproduction and survival.

## Author Contributions

**Ryan W. Koch:** conceptualization (equal), data curation (lead), formal analysis (lead), investigation (equal), methodology (equal), writing – original draft (lead). **Axel O. G. Hoarau:** investigation (equal), methodology (equal), writing – review and editing (equal). **Tryssa de Ruyter:** investigation (equal), methodology (equal), writing – review and editing (equal). **Caitlin Duffy:** investigation (equal), methodology (equal). **Lucie Pascarosa:** investigation (equal), methodology (equal), writing – review and editing (equal). **Kerry A. Campbell:** investigation (equal). **Casey L. Maynard:** investigation (equal), writing – review and editing (equal). **Andrew Cushman:** investigation (equal). **Heather Flick:** investigation (equal). **Anthony Musselman:** investigation (equal). **Julianna Patsko:** investigation (equal). **Rachel Bealer:** investigation (equal). **Graham Rhone:** investigation (equal). **Mary Jo Casalena:** investigation (equal), methodology (equal), supervision (equal), validation (equal), writing – review and editing (equal). **Andrew Di Salvo:** methodology (equal), validation (equal), writing – review and editing (equal). **Ken Duren:** conceptualization (equal), funding acquisition (equal), validation (equal), writing – review and editing (equal). **Jay T. Armstrong:** investigation (equal). **Frances E. Buderman:** validation (equal), writing – review and editing (equal). **R. Scott Larsen:** methodology (equal), validation (equal), writing – review and editing (equal). **Caroline Sobotyk:** methodology (equal), supervision (equal), validation (equal), writing – review and editing (equal). **Erica A. Miller:** methodology (equal), supervision (equal), validation (equal), writing – review and editing (equal). **Kevin D. Niedringhaus:** validation (equal), writing – review and editing (equal). **Brock Geary:** data curation (equal), formal analysis (supporting), validation (equal), writing – review and editing (equal). **Eman Anis:** investigation (equal), methodology (equal), supervision (equal), writing – review and editing (equal). **Roderick B. Gagne:** conceptualization (equal), funding acquisition (equal), investigation (equal), methodology (equal), supervision (equal), writing – original draft (equal).

## Funding

This work was supported by the Richard King Mellon Foundation (Grant 11494) and Pennsylvania Game Commission (Grant 4000029063).

## Conflicts of Interest

The authors declare no conflicts of interest.

## Supporting information


**Table S1:** List of models run in analyzes and associated variables.

## Data Availability

The data from this study are available at: https://doi.org/10.5061/dryad.n2z34tnbk.
