# Supplementary material for: Risk Factors and Coinfection Dynamics of Pathogens in Wild Turkeys (Meleagris gallopavo) From Pennsylvania, USA
Source: Ecol Evol. 2026 Feb 10;16(2):e73079. doi: 10.1002/ece3.73079 (PMC12891437; doi:10.1002/ece3.73079)
Supplement: Supplementary file 1 — Table S1: List of models run in analyzes and associated variables. [file ECE3-16-e73079-s001.docx]

| Model | Response variable (y) | Predictor variable (x) |
| --- | --- | --- |
| 1. Linear regression | Group capture size | LPDV group capture prevalence |
| 2. Mixed effects logistic regression | LPDV infection | Age, sex, year, study area, landscape type, capture event (random effect) |
| 3. Poisson regression | Parasite species richness | LPDV, age, sex, year, study area, and landscape type |
| 4. Logistic regression | Parasite: *Capillaria* sp. | LPDV, age, sex, year, study area, and landscape type |
| 5. Logistic regression | Parasite: *Eimeria* sp. | LPDV, age, sex, year, study area, and landscape type |
| 6. Logistic regression | Parasite: Ascarid | LPDV, age, sex, year, study area, and landscape type |
| 7. Logistic regression | Coinfection | Age, sex, year, study area, landscape type |
| 8. Linear regression | Parasite county prevalence | LPDV county prevalence |

Table S1. List of models run in analyses and associated variables.
